# Mortality and cancer incidence in UK military veterans involved in human experiments at Porton Down: 48-year follow-up

**DOI:** 10.1093/ije/dyad050

**Published:** 2023-05-11

**Authors:** Gemma Archer, Thomas J Keegan, Lucy M Carpenter, Katherine M Venables, Nicola T Fear

**Affiliations:** King’s Centre for Military Health Research, Department of Psychological Medicine, King’s College London, London, UK; Lancaster Medical School, Lancaster University, Lancaster, UK; Nuffield College, University of Oxford, Oxford, UK; Nuffield Department of Population Health, University of Oxford, Oxford, UK; King’s Centre for Military Health Research, Department of Psychological Medicine, King’s College London, London, UK

**Keywords:** Veterans, military health, cohort studies, mortality, cancer incidence, chemical exposure, toxicology

## Abstract

**Background:**

We investigated whether military personnel involved in chemical warfare agent research at Porton Down had increased rates of mortality or cancer incidence.

**Methods:**

This was a historical cohort study comprising male UK veterans who participated in the ‘Service Volunteer Programme’, 1941–89, identified from Porton Down experiment books, and a comparison group of similar ‘non-Porton Down’ veterans identified from military personnel files. Of 19 233 records retrieved for each group, 18 133 (94%) Porton Down and 17 591 (92%) non-Porton Down were included in our analytical sample. Mortality and cancer incidence data were obtained from national registries up to December 2019.

**Results:**

Over a median follow-up of 48.1 years, 10 935 Porton Down veterans (60.3%) and 10 658 non-Porton Down veterans (60.6%) had died. After adjustment for age, year of birth and military service characteristics, overall, Porton Down veterans had a 6% higher rate of all-cause mortality compared with non-Porton Down veterans [hazard ratio (HR) = 1.06, 95% confidence interval (CI) 1.03–1.09]. For cause-specific mortality, Porton Down veterans had higher rates of death from genitourinary diseases (HR = 1.34, 95% CI 1.05–1.70) and deaths attributable to alcohol (HR = 1.44, 95% CI 1.07–1.94), with weaker associations observed for deaths from infectious and parasitic diseases (HR = 1.32, 95% CI 0.99–1.78), lung cancer (HR = 1.10, 95% CI 1.01–1.20) and external causes (HR = 1.15, 95% CI 1.00–1.32). Associations with all-cause mortality were stronger for veterans who attended Porton Down between 1960 and 1964 (HR = 1.34, 95% CI 1.19–1.50); likelihood-ratio test, *P *= 0.006. There was no association between attendance at Porton Down and overall cancer incidence (HR = 1.00, 95% CI 0.95–1.03).

**Conclusions:**

Overall, mortality rates were slightly higher in Porton Down veterans, but there was no difference in cancer incidence. Associations for mortality were stronger in Porton Down veterans who attended in the early 1960s.

Key Messages

- Little is known about the long-term health effects of low-dose exposure to chemical warfare agents.
- UK military personnel who were involved in chemical warfare agent research at Porton Down between 1941 and 1989 were followed up for mortality and cancer incidence.
- Porton Down veterans had a slightly higher (6%) rate of all-cause mortality compared with non-Porton Down veterans, and there was no difference in cancer incidence; however, hazard ratios were raised for some specific causes of death and types of cancer.
- Associations for mortality were stronger in Porton Down veterans who attended in the early 1960s, compared with other attendance periods.
- Health providers should be aware of the specific health issues and concerns surrounding military personnel and others (e.g. civilians and emergency responders) who may have experienced exposure to chemical warfare agents.

## Introduction

In response to the use of chemical weapons during World War I, the UK government began human and animal research at Porton Down, UK, to learn more about the development and effects of these agents and protective clothing and medical countermeasures. Since 1916, over 20 000 military service personnel have attended Porton Down as part of the ‘Service Volunteer Programme’,^1^ during which many service personnel were exposed to low doses of chemical warfare agents and their antidotes, some of which are known to be carcinogenic.^2^

The Porton Down Cohort Veterans Study was assembled in the early 2000s in response to concerns raised by former UK service personnel that their long-term physical and psychological health may have been affected by their involvement in the programme. Findings from the first phase of the study, which followed mortality and cancer incidence up to December 2004, found that Porton Down veterans had a 6% higher all-cause mortality rate but similar cancer incidence compared with non-Porton Down veterans.^3^^,^^4^ Despite similar programmes of chemical warfare agent testing in, for example, the USA, Japan and Australia, follow-up studies are scarce and have demonstrated mixed results.^5–7^

The Porton Down Veterans Cohort Study is the largest of its kind, and recently underwent a second phase of data linkage to include an additional 15 years of follow-up data.^8^ Our aim is to use these updated data to re-examine whether attendance at Porton Down is associated with increased rates of mortality or cancer incidence, compared with veterans who did not attend.

## Methods

### Assembly of the cohort

The Porton Down Veterans cohort is a retrospective cohort study that comprises two sub-cohorts of military veterans: the ‘Porton Down veterans’—all those identified from historical records at Porton Down as having participated in the Service Volunteer Programme between 1 April 1941 and 31 December 1989; and ‘non-Porton Down veterans’—a comparison cohort of veterans who did not attend Porton Down and who were identified by selecting the adjacent service number (either above and below) of every Porton Down veteran. Military characteristics and demographic information were abstracted from military personnel archives. Further detail on assembly of the Porton Down Veteran and comparison cohorts can be found elsewhere.^3^^,^^4^

### Data flow and linkage

Supplementary Figure S1 (available as Supplementary data at *IJE* online) shows 19 233 valid records were identified for both Porton Down and non-Porton Down veterans; 18 466 (96.0%) and 18 129 (94.2%), respectively, were successfully retrieved and contained sufficient information for linkage with National Health Service (NHS) Central Registry data. Of these records, 16 721 (90.5%) Porton Down and 16 228 (89.5%) non-Porton Down veterans were successfully traced.

### Mortality and cancers

Information on mortality and emigration was obtained from the National Health Service Central Register (NHSCR). For untraced veterans, additional deaths were identified from the Commonwealth War Graves Commission, Department for Work and Pensions and military personnel records (5.7%).

Cancer site and date of registration were available from national cancer registries from 1 January 1971. For cancers listed on the death certificate only, we used date of death as the date of cancer registration (n = 438, 4.0%).

Cause of death and cancer registrations were coded according to ICD-10 (International Classification of Diseases, 10th revision).^9^ We focused on the most common causes of death and types of cancer; other outcomes of interest were identified a priori based on plausible mechanisms and associations demonstrated in existing literature.^2–4^^,^^10^^,^^11^

#### Follow-up

For mortality, person-years of follow-up for Porton Down veterans started from the date of their first test at Porton Down (or date of arrival at Porton Down if missing; *n* = 974). Follow-up time for mortality was until date of death or 31 December 2019, or was censored at the last known date alive (i.e. date of emigration, discharge from the services or end of the first phase of follow-up if previously traced).

For cancer analysis, person-years of follow-up for Porton Down veterans started from 1 January 1971 or the date of first test at Porton Down—whichever came later. Follow-up for cancer was until date of first cancer registration or until 31 December 2019, or was censored at death or date last known alive.

Start of follow-up for non-Porton Down veterans was derived by adding to the enlistment date the interval between the corresponding Porton Down veteran’s dates of enlistment and start of follow-up.

### Confounding factors

Confounders were identified as factors potentially associated with both exposure and outcome; these included age at exposure/start of follow-up (years), year of birth, service at enlistment (Army, Air Force, Royal Navy, Marines), previous duration of service (years), period of enlistment (before, during and after World War II, and post-National Service), and place of birth (England, Scotland, Wales, Northern Ireland, other).

### Study sample

Eligible cohort members were all UK servicemen who participated in the Service Volunteer Programme at Porton Down between 1 April 1941 and 31 December 1989, and their corresponding non-Porton Down veteran, who had complete data on mortality, start and end person-years of follow-up and covariates.

In all, 18 441 Porton Down veterans and 17 846 non-Porton Down veterans were successfully traced or had first-phase study data; exclusions (308 Porton Down veterans and 255 non-Porton Down veterans) included those with missing or implausible data (e.g. veterans who were <14 years old at enlistment, veterans who died prior to their date of enlistment) and a small number of women (*n *= 132) (Supplementary Figure S1, available as Supplementary data at *IJE* online). The analytical sample for mortality consisted of 18 133 Porton Down and 17 591 non-Porton Down veterans.

Prior to the start of follow-up for cancers (1 January 1971), 849 Porton Down veterans and 761 non-Porton Down veterans had died and 357 Porton Down veterans and 295 non-Porton Down veterans were lost to follow-up. The analytical sample for cancer incidence consisted of 16 927 Porton Down veterans and 16 533 non-Porton Down veterans.

### Statistical analysis

Kaplan–Meier graphs were used to compare the survival probability of those who did and did not attend Porton Down.

All-cause and cause-specific mortality rates between Porton Down and non-Porton Down veterans were compared using Cox’s proportional hazards models, with time since start of follow-up as the time scale. Models were first adjusted for age (at start of follow-up) and calendar period (year of birth); and then further adjusted for military demographics (branch of service, previous duration of service, period of joining and place of birth). Since experimental programmes at Porton Down changed over time, we also examined associations between period of Porton Down attendance and mortality by 5-year bands; interactions were tested using likelihood ratio tests. We also conducted exploratory analysis examining whether specific chemical exposures to which at least 100 veterans were exposed accounted for the excess mortality observed in veterans who attended Porton Down between 1960 and 1964. Deaths 100% attributable to alcohol were defined by ICD-10 codes F102, K701, K703, K704, K709^12^, and deaths strongly causally related to smoking by ICD-10 codes C33, C34, J40-J44.^13^

We calculated E-values for both the observed association estimates and the limit of the confidence interval closest to the null to assess the robustness of associations between Porton Down attendance and outcome variables.^14^

Competing risk analysis provided similar but slightly attenuated estimates for key associations (Supplementary Results 1, available as Supplementary data at *IJE* online).

The proportional hazards assumption was tested using Schoenfeld residuals; the assumption was violated by age and calendar period (*P *<0.05), so baseline hazard functions for these variables were allowed to vary by stratum (5-year bands).

The above analyses were repeated for cancer incidence outcomes as appropriate. All analyses were conducted using STATA v16.1 (StataCorp, 2019).

## Results

### Descriptive characteristics

Descriptive characteristics for Porton Down veterans and non-Porton Down veterans were largely identical except for ‘total duration of service’; Porton Down veterans tended to serve for longer. For example, 27.8% of Porton Veterans served over 10 years, compared with 17.7% of non-Porton Down Veterans (Supplementary Table S1, available as Supplementary data at *IJE* online). The majority of veterans served in the Army, were born in England before 1940, and enlisted in their teenage years under periods of conscription, either during World War II (1939–45) or during ‘National Service’ (1945–60). Descriptive characteristics for veterans with complete data on cancer registrations were similar (Supplementary Table S2, available as Supplementary data at *IJE* online).

### All-cause mortality

**Figure 1:**
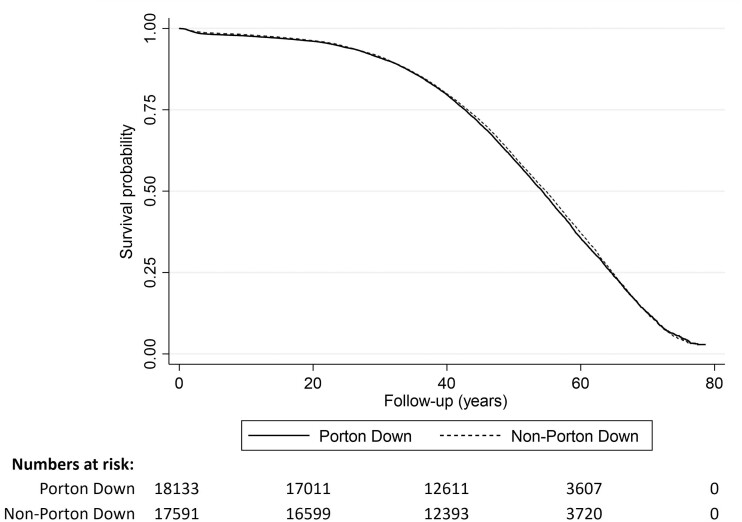
Unadjusted Kaplan–Meier survival estimates for all-cause mortality by Porton Down attendance

A total of 10 967 Porton Down veterans and 10 660 non-Porton Down veterans died over a median follow-up of 48.1 years (range 2 days to 78.7 years). Incidence rates for all-cause mortality were 13.0 per 1000 person-years for Porton Down veterans and 12.9 per 1000 person-years for non-Porton Down veterans. Kaplan–Meier survival curves show that Porton Down veterans and non-Porton Down veterans had comparable survival probabilities over time (Figure 1).

Across all periods of attendance and after full adjustment, Porton Down veterans had a slightly higher mortality rate compared with non-Porton Down veterans [hazard ratio (HR)=1.06, 95% confidence interval (CI) 1.03–1.09] (Table 1).

**Table 1:** Hazard ratios for the association between attendance at Porton Down and cause-specific mortality in 18 069 Porton Down veterans and 17 588 non-Porton Down veterans

	Observed deaths (*n*)		Hazard ratios (95% CI)			
Cause of death (ICD-10 code)	Porton Down veterans	Non-Porton Down veterans	Adjusted for age and calendar period	Fully adjusted^a^	Fully adjusted^a^ (excluding 1960-1964 attendance)	Fully adjusted^a^ (1960-1964 attendance only)
All-cause	10 935	10 658	1.06 (1.03–1.09)	1.06 (1.03–1.09)	1.04 (1.02-1.07)	1.34 (1.19-1.50)
Infectious and parasitic (A00-B99)	103	80	1.33 (0.99–1.78)	1.32 (0.99–1.78)	1.27 (0.94-1.71)	4.26 (0.80-2.74)
Malignant neoplasms (All) (C00-97)	3247	3199	1.05 (1.00–1.10)	1.05 (1.00–1.10)	1.02 (0.97-1.08)	1.47 (1.21-1.78)
Upper aerodigestive (C00-14, C30-32)	91	79	1.16 (0.86–1.57)	1.16 (0.86–1.57)	1.19 (0.86-1.62)	0.89 (0.30-2.68)
Oesophageal (C15)	156	167	0.95 (0.76–1.18)	0.95 (0.76–1.18)	0.89 (0.71-1.12)	2.44 (1.04-5.74)
Lung (C34)	1116	1032	1.11 (1.02–1.21)	1.10 (1.01–1.20)	1.08 (0.99-1.18)	1.68 (1.15-2.46)
Brain and other central nervous system (C71, C72)	63	85	0.72 (0.52–1.00)	0.73 (0.52–1.01)	0.68 (0.48, 0.96)	1.22 (0.49-3.02)
All lymphatic and haematopoietic (C81-96)	215	239	0.92 (0.77–1.11)	0.92 (0.77–1.11)	0.92 (0.76-1.12)	0.89 (0.43-1.87)
*In situ*, benign, and unspecified neoplasms (D10-48)	50	76	0.69 (0.48–0.98)	0.70 (0.49–1.00)	0.70 (0.48-1.02)	0.66 (0.16-2.80)
Nervous system (G00-99)	247	282	0.92 (0.78–1.10)	0.93 (0.78–1.10)	0.97 (0.81-1.15)	0.50 (0.24-1.06)
Circulatory system, all (I00-99)	4064	3955	1.06 (1.01–1.11)	1.06 (1.01–1.10)	1.04 (1.00-1.09)	1.38 (1.12-1.69)
Ischaemic heart diseases (I20-25)	2618	2490	1.08 (1.02–1.14)	1.08 (1.02–1.14)	1.05 (1.00-1.11)	1.60 (1.24, 2.06)
Cerebrovascular diseases (I60-69)	710	736	1.01 (0.91–1.12)	1.00 (0.90–1.11)	1.00 (0.90-1.11)	1.07 (0.62-1.86)
All other circulatory (I00-19, I26-59, I70-99)	736	729	1.05 (0.94–1.16)	1.05 (0.94–1.16)	1.05 (0.95-1.17)	1.01 (0.63-1.62)
Respiratory system, all (J00-99)	1210	1240	1.03 (0.95–1.11)	1.03 (0.95–1.11)	1.02 (0.94-1.10)	1.32 (0.90-1.95)
Chronic lower respiratory tract (J40-47)	689	692	1.03 (0.92–1.14)	1.03 (0.92–1.14)	1.02 (0.91-1.13)	1.19 (0.72-1.94)
All other respiratory (J00-39, J48-99)	521	548	1.02 (0.91–1.16)	1.03 (0.91–1.16)	1.01 (0.90-1.15)	1.61 (0.86-3.03)
Genitourinary system (N00-99)	149	116	1.33 (1.04–1.70)	1.34 (1.05–1.70)	1.39 (1.08-1.78)	0.71 (0.23-2.22)
All external causes (S00-T98, V01-Y98)	428	371	1.15 (1.00–1.32)	1.15 (1.00–1.32)	1.13 (0.97-1.30)	1.53 (0.88-2.64)
Alcohol-attributable (F102, K701, K703, K704, K709)	108	76	1.42 (1.06–1.90)	1.44 (1.07–1.94)	1.47 (1.08-2.00)	1.17 (0.44-3.06)
Smoking-related (highly causal only; C33, C34, J40-J44)	1774	1691	1.07 (1.03–1.10)	1.07 (1.01–1.15)	1.06 (0.99-1.13)	1.48 (1.09-2.00)

Reference category: ‘Non-Porton Down veterans’.

ICD-10: International Classification of Diseases 10th Revision.^9^

a Adjusted for branch of service, previous duration of service, period of joining and place of birth.

**Figure 2:**
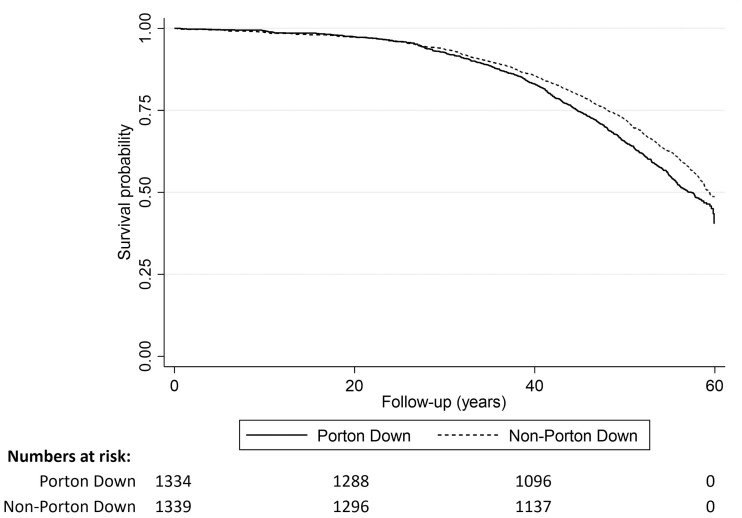
Unadjusted Kaplan–Meier survival estimates for all-cause mortality for veterans attending Porton Down between 1960 and 1964 only

We observed stronger associations between Porton Down veterans and mortality among those who attended between 1960 and 1964 compared with other attendance periods (fully adjusted HR = 1.34, 95% CI 1.19–1.50; likelihood ratio test, *P *= 0.006) (Supplementary Table S3, available as Supplementary data at *IJE* online). This association was not driven by any particular year between 1960 and 1964. Notably, Kaplan–Meier survival curves for Porton Down and non-Porton Down veterans only began to diverge, indicating differing rates of mortality, after approximately 25 years of follow-up (Figure 2). Exploratory analyses demonstrated that age-adjusted hazards ratios were raised for multiple types of chemical exposure during this period (Supplementary Results S2, available as Supplementary data at *IJE* online). Veterans were often exposed to more than one type of chemical (for example, half of veterans exposed to 2-chlorobenzalmalononitrile (CS) gas, pralidoxime, sarin or dibenzoxazepine (CR) gas were also exposed to sulphur mustard) so it was not possible to identify whether excess mortality was driven by a specific exposure(s).

### Cause-specific mortality

Across all periods of attendance, Porton Down veterans had higher rates of mortality from diseases of the genitourinary system (fully adjusted HR = 1.34, 95% CI 1.05–1.70) and deaths attributable to alcohol (HR = 1.44, 95% CI 1.07–1.94) compared with non-Porton Down veterans (Table 1). Weaker associations were observed for deaths from infectious and parasitic diseases (HR = 1.32, 95% CI 0.99–1.78), malignant neoplasms (HR = 1.05, 95% CI 1.01–1.10), lung cancers (HR = 1.10, 95% CI 1.01–1.20), diseases of the circulatory system (HR = 1.06, 1.01–1.11), external causes (e.g. accidental and violent deaths or suicide; HR = 1.15, 95% CI 1.00–1.32) and smoking-related deaths (HR = 1.07, 95% CI 1.01–1.15). Conversely, Porton Down veterans had lower rates of mortality from ‘brain and other central nervous system neoplasms’ (HR = 0.71, 95% CI 0.51–0.99) and *in situ* benign and unspecified neoplasms (HR = 0.70, 95% CI 0.49–1.00), compared with non-Porton Down veterans. Most associations were unaffected by excluding those who attended Porton Down between 1960 and 1964.

In contrast to other attendance periods, veterans who attended Porton Down between 1960 and 1964 demonstrated stronger associations with death from: any malignant neoplasm (HR = 1.47, 95% CI 1.21–1.78), oesophageal cancers (HR = 2.44, 95% CI 1.04–5.74) and lung cancers (HR = 1.68, 95% CI 1.15–2.46); all diseases of the circulatory system (HR = 1.38, 95% CI 1.12–1.69) and ischaemic heart disease (HR = 1.60, 95% CI 1.24–2.06); and smoking related deaths (HR = 1.48, 95% CI 1.09–2.00).

### Cancer incidence

Over a median follow-up of 34.0 years (range 1 day to 49.0 years), 5396 (31.9%) of Porton Down veterans and 5475 (33.1%) non-Porton Down veterans had at least one cancer (Supplementary Table S2, available as Supplementary data at *IJE* online). Cancer incidence rates were almost identical: 10.0 per 1000 person-years for Porton Down veterans and 10.3 per 1000 person-years for non-Porton Down veterans, with Kaplan–Meier plots indicating similar survival probabilities over follow-up (Supplementary Figure S2, available as Supplementary data at *IJE* online).

In fully adjusted models, there was no evidence of an association between Porton Down attendance and overall incidence of any neoplasm (HR = 1.00, 95% CI 0.96–1.03) or malignant neoplasms (HR = 0.99, 95% CI 0.95–1.03); however, Porton Down veterans did have higher rates of neoplasms of ‘uncertain or unknown behaviour’ (HR = 1.27, 95% CI 1.06–1.54). Weaker associations were observed for neoplasms of the trachea, bronchus and lung (HR = 1.09, 95% CI 1.00–1.18) and all other primary malignant neoplasms (HR = 1.19, 95% CI 1.00–1.42). Porton Down attendance was also associated with lower rates of ‘other skin’ neoplasms (HR = 0.90, 95% CI 0.82–0.98) and ‘other urinary tract’ neoplasms (HR = 0.79, 95% CI 0.62–1.00) (Table 2). There was no evidence that these associations differed by period of attendance (likelihood ratio test, *P *= 0.41).

**Table 2:** Hazard ratios for the association between Porton Down attendance and cancer incidence in 16 927 Porton Down veterans and 16 533 non-Porton Down veterans

	Observed cases (*n*)		Hazard ratios (95% CI)	
Cancer site/type (ICD-10 code)	Porton Down veterans	Non-Porton Down veterans	Adjusted for age and calendar period	Fully adjusted^a^
Any neoplasm (C00-C97, D00-D48)	5396	5475	1.00 (0.96–1.03)	1.00 (0.96–1.03)
Any malignant neoplasm (C00-C97)	5124	5225	0.99 (0.95–1.03)	0.99 (0.95–1.03)
Upper aerodigestive (C00-C14, C30-C32)	213	186	1.14 (0.94–1.39)	1.15 (0.94–1.40)
Oesophagus (C15)	160	160	1.02 (0.82–1.27)	1.02 (0.82–1.27)
Stomach (C16)	226	222	1.04 (0.86–1.25)	1.03 (0.86–1.24)
Intestine and rectum (C17-C20)	622	629	1.01 (0.91–1.13)	1.01 (0.91–1.13)
Pancreas (C25)	140	129	1.13 (0.89–1.44)	1.13 (0.89–1.44)
Trachea, bronchus and lung (C33, C34)	1203	1125	1.09 (1.00–1.18)	1.09 (1.00–1.18)
Melanoma of skin (C43)	108	105	1.05 (0.80–1.38)	1.05 (0.80–1.37)
Other skin (C44)	1069	1201	0.91 (0.84–0.99)	0.90 (0.82–0.98)
Prostate (C61)	843	917	0.94 (0.86–1.04)	0.95 (0.86–1.04)
Bladder (C67)	280	318	0.91 (0.77–1.07)	0.90 (0.77–1.06)
Other urinary tract (C64-C66, C68)	121	155	0.79 (0.62–1.00)	0.79 (0.62–1.00)
Brain and other central nervous system (C71, C72)	71	92	0.77 (0.56–1.04)	0.77 (0.57–1.06)
All leukaemias (C91-C95)	122	151	0.83 (0.65–1.05)	0.83 (0.65–1.05)
Other lymphatic and haematopoietic (C81-C90, C96)	231	228	1.03 (0.86–1.23)	1.03 (0.86–1.23)
Ill-defined, secondary, or unspecified malignant neoplasms (C76-C80)	221	214	1.05 (0.87–1.27)	1.06 (0.88–1.28)
All other primary malignant neoplasms^b^	271	233	1.19 (1.00–1.42)	1.19 (1.00–1.42)
Alcohol-related neoplasms^c^	1016	979	1.06 (0.97–1.16)	1.06 (0.97–1.16)
Smoking-related neoplams^d^	2927	2864	1.04 (0.99–1.10)	1.04 (0.99–1.09)
Any *in situ* neoplasm (D00-D09)	267	242	1.14 (0.96–1.36)	1.14 (0.96–1.36)
Any benign neoplasm (D10-D36)	40	45	0.89 (0.58–1.36)	0.89 (0.58–1.37)
Any neoplasm of uncertain or unknown behaviour (D37-D48)	244	198	1.27 (1.05–1.53)	1.27 (1.06–1.54)

Reference category: ‘Non-Porton Down veterans’.

ICD-10: International Classification of Diseases 10th Revision.^9^

a Adjusted for branch of service, previous duration of service, period of joining and place of birth.

b Other primary malignant neoplasms (C21-C24, C26, C7, C38, C40, C41, C45, C47-50, C53, C54, C56, C60, C62, C63, C69, C70, C73-75).

c Alcohol-related neoplasms (C01-6, C09-10, C12-13, C15, C18, C19-20, C22, C32, C50).

d Smoking-related neoplasms (C00–16, C18-20, C22, C25, C32-34, C53, C64-C67, C92).

### E-values

Across all periods of attendance, the E-value for the fully adjusted association between Porton Down attendance and all-cause mortality was 1.25 and the E-value for the limit of the confidence interval closest to the null (E_CI_) was 1.17 (Supplementary Table S4, available as Supplementary data at *IJE* online). Larger E-values were observed for deaths from diseases of the genitourinary system (2.02, E_CI_ 1.28) and alcohol attributable deaths (2.24, E_CI_ 1.34). For attendance between 1960 and 1964, the E-value for all-cause mortality was 1.75 (E_CI_ 1.51), with E-values ranging between 1.94 and 4.31 (E_CI_ range 1.40–1.79) for deaths from malignant neoplasms, oesophageal and lung cancers, diseases of the circulatory system, ischaemic heart disease and smoking-related deaths. For cancer incidence, the E-values for ‘any neoplasm of uncertain or unknown behaviour’ were 1.86 (E_CI_ 1.31).

## Discussion

Across all periods of attendance, Porton Down veterans (*n* = 18 133) had a 6% higher relative rate of all-cause mortality compared to non-Porton Down veterans (*n* = 17 591). For cause-specific mortality, Porton Down attendance was associated with higher rates of death from diseases of the genitourinary system (34%) and alcohol-attributable deaths (44%). Weaker associations were observed for deaths from infectious and parasitic diseases (32%), malignant neoplasms (5%), lung cancers (10%), circulatory diseases (6%), external causes (15%) and smoking-related deaths (7%). We found that the association between Porton Down attendance and mortality was stronger in veterans who attended Porton Down between 1960 and 1964 compared with other periods. Attendance during the early 1960s was associated with a higher rate of all-cause mortality (34%), deaths from malignant neoplasms (47%), diseases of the circulatory system (38%) and smoking related deaths (48%) compared with non-Porton Down veterans. There was little evidence of an association between Porton Down attendance and higher rates of overall cancer incidence, nor for most types of cancer.

The Porton Down Veterans cohort study comprises a large group of older veterans who served in the UK military. The cohort benefits from a long follow-up, spanning several decades. The study relies on historical records and national registry data, so has low levels of attrition and potential for selection bias; however for cancer analyses, there is a risk of survivor bias and we cannot rule out chance findings due to testing numerous exposure-outcome associations. Another limitation of the cohort is that there were too few women to examine sex differences, and we had no information on potential confounders including combat exposure, smoking, alcohol and socioeconomic factors. E-value calculations suggested that weaker associations between Porton Down attendance and mortality and cancers were vulnerable to potential unmeasured confounding but that other associations were relatively robust. For example, the fully adjusted hazard ratio for ‘alcohol-attributable deaths’ (HR = 1.44, 95% CI 1.07–1.94) could only be accounted for by an unmeasured confounder associated with both the exposure and outcome by a risk ratio of at least 2.24, and the confidence interval could be moved to include the null by an unmeasured confounder with a risk ratio of at least 1.34, above and beyond the measured confounders. We have no evidence that the prevalence of unmeasured confounders should be considerably higher in veterans who attended Porton Down compared with non-Porton Down veterans, as they were matched by the time and location of recruitment. Further, if the prevalence of, for example, smoking, alcohol misuse and social disadvantage were markedly higher in veterans who attended Porton Down, we would expect to see excess mortality across numerous outcomes typically associated with these factors.^15^

Our results are consistent with those reported in the first phase of the Porton Down Veterans Cohort study, which followed veterans for mortality and cancer incidence until December 2004.^3^^,^^4^ Analysis from the first phase did not show higher rates of mortality in veterans who attended Porton Down in the early 1960s becasue this association only became apparent after approximately 2 decades of follow-up.

Several smaller US studies have examined the long-term health of military personnel who participated in chemical warfare agent research. Project SHAD (Shipboard Hazard and Defence) veterans (*n *= 4487), exposed to chemical and biological agents, were found to have higher rates of all-cause mortality [rate ratio (RR) = 1.10, 95% CI 1.01–1.97] and deaths from heart disease (RR = 1.39, 95% CI 1.18–1.64) compared with non-SHAD veterans, yet no associations were reported for other causes of death, including deaths from infectious and parasitic diseases (HR = 0.66, 95% CI 0.38–1.14) and external causes (HR = 0.79, 95% CI 0.61–1.03).^6^ These discrepancies are likely explained by differences in the type of exposures, poor-quality exposure data^6^ and fewer deaths (leading to less precise estimates). Null associations were also found between 4826 veterans exposed to multiple chemical agents during participation in trials at US Army Laboratories in Edgewood, Maryland (USA) and several long-term health outcomes; this study was vulnerable to selection bias as it was reliant on self-report questionnaire and the pre-existing health of each veteran determined the type of test in which they participated. Similarly, no associations were found between 1545 US Navy veterans who participated in mustard gas chamber tests in Bainbridge, Maryland (USA), and all-cause mortality, deaths from cancer or other causes. Since there is good evidence to suggest mustard gas is carcinogenic,^10^^,^^17^ the authors attributed this to participants wearing protective equipment and being exposed at low doses for short periods of time.

There are several possible explanations for excess mortality observed in Porton Down veterans. For example, potentially causal relationships have been indicated between mustard gas exposure and bone marrow depression and immunosuppression,^10^ and Lewisite exposure and acute kidney injury^18^—suggestive of possible pathways from chemical exposure to deaths from infectious and parasitic and genitourinary diseases. Higher rates of alcohol-attributable deaths (and to a lesser extent deaths from external causes) in Porton Down veterans could imply psychological injury, with high rates of post-traumatic stress disorder (PTSD) reported in US veterans who participated in mustard gas trials.^19^

Higher rates of deaths from cancers, and circulatory diseases observed in Porton Down veterans who attended in the early 1960s (*n* = 1175) could be explained if Porton Down veterans who attended during this time were systematically different in some way due to their recruitment. Conversely, these results could suggest that their chemical exposures were unusual during this period, whether by dose or by type. Nearly 5000 tests involving 44 different chemicals were conducted during 1960 to 1964; it was not possible to establish whether excess mortality during this period could be ascribed to a single type of chemical(s) as exploratory analyses demonstrated that hazard ratios were raised for most chemicals to which at least 100 veterans were exposed, and veterans were often exposed to multiple types of chemical, making it difficult to establish whether excess mortality was driven by a specific exposure(s).

## Conclusion

Overall, our findings and those of other studies^5^^,^^6^^,^^10^^,^^20^ indicate that the large majority of veterans who participated in chemical warfare agent research programmes are unlikely to have come to harm. We did find that a small number of Porton Down veterans had higher rates of specific causes of death and types of cancer. It is not possible to causally attribute this to chemical exposure; however, health providers should be aware of the health issues and concerns surrounding veterans, their families and other civilian, emergency responder or military personnel who may have experienced exposure to chemical agents.

## Ethics approval

For England and Wales, ethics approval for the most recent phase of the study was granted by the Research Ethics Service Committee (14/LO/1760) and the Health Research Authority’s Confidentiality Advisory Group (CAG; 18/CAG/0171) under section 251 of the National Health Services Act 2006. For Scotland, approval was granted by the Public Benefit and Privacy Panel for Health and Social Care (PBPP-HSC). Data Sharing Agreements are in place with NHS Digital and the PBPP-HSC, which are reviewed annually.

## Supplementary Material

dyad050_Supplementary_Data

## Data Availability

This work uses data provided by patients and collected by the NHS as part of their care and support. Mortality and cancer registry data are provided by permission of NHS Digital and the Public Benefit and Privacy Panel for Health and Social Care. The cohort data are not freely available due to legal and ethical restrictions in place to protect the privacy of research participants; however, the study team welcomes enquiries for research proposals and collaboration. Interested parties should contact the study lead, Professor Nicola Fear [nicola.t.fear@kcl.ac.uk] who will be able to advise on feasibility and necessary permissions.
