# Supplementary material for: Mortality and cancer incidence in UK military veterans involved in human experiments at Porton Down: 48-year follow-up
Source: Int J Epidemiol. 2023 May 11;52(4):1025–34. doi: 10.1093/ije/dyad050 (PMC10396403; doi:10.1093/ije/dyad050)
Supplement: dyad050_Supplementary_Data [file dyad050_supplementary_data.docx]

**Supplementary material**

**Figure S1 2**

**Figure S2 3**

**Table S1 4**

**Table S2 6**

**Tabe S3 8**

**Table S4 9**

**Supplementary results 1 11**

**Supplementary results 2 11**


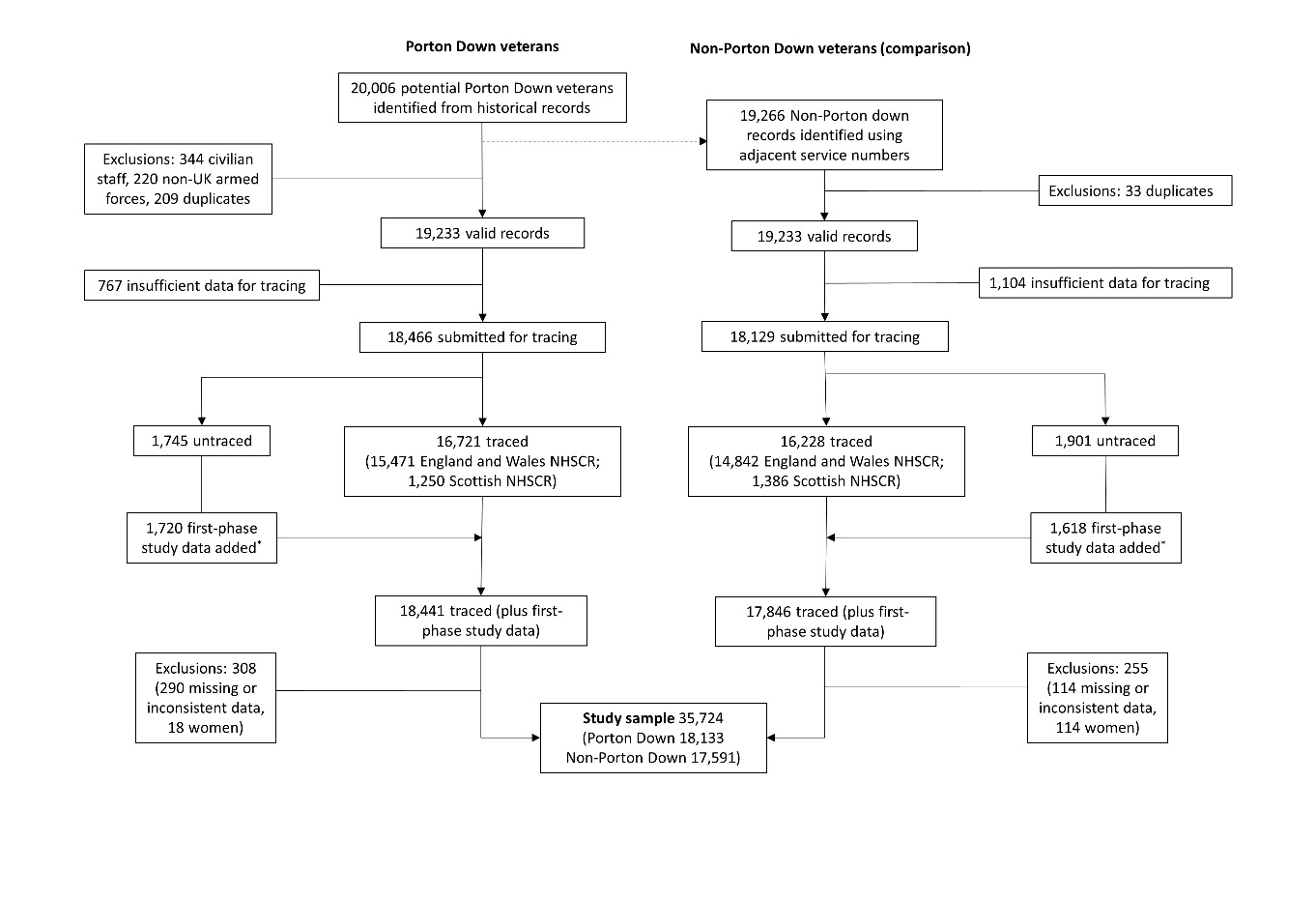


**Figure S1**. Participant flow

**Supplementary figure S2.** Unadjusted Kaplan Meier survival estimates for all neoplasm incidence by Porton down attendance

**Table S1.** Characteristics of the study sample comprising 18,133 Porton Down veterans and 17,591 non-Porton Down veterans

|  | **Porton Down veterans** | **Non-Porton Down veterans** |
| --- | --- | --- |
|  | n (%) | n (%) |
| **Service at enlistment** |  |  |
| Army | 11,292 (62.3) | 10,843 (61.6) |
| Airforce | 4,009 (22.1) | 3,991 (22.7) |
| Navy | 2,832 (15.6) | 2,757 (15.7) |
| **Decade of birth** |  |  |
| 1920< | 3,647 (20.1) | 3,746 (21.3) |
| 1920-29 | 3,505 (19.3) | 3,218 (18.3) |
| 1930-39 | 6,075 (33.5) | 5,990 (34.1) |
| >1940 | 4,906 (27.1) | 4,637 (26.4) |
| **Place of birth** |  |  |
| England | 14,202 (78.3) | 13,737 (78.1) |
| Scotland | 1,758 (9.7) | 1,821 (10.4) |
| Wales | 925 (5.1) | 917 (5.2) |
| Northern Ireland | 303 (1.7) | 248 (1.4) |
| Other | 945 (5.2) | 868 (4.9) |
| **Age at enlistment (years)** |  |  |
| <16 | 1,237 (6.8) | 1,225 (7.0) |
| 16-17 | 4,986 (27.5) | 4,310 (24.5) |
| 18-19 | 6,486 (35.8) | 6,221 (35.4) |
| 20-21 | 2,264 (12.5) | 2,354 (13.4) |
| >21 | 3,160 (17.4) | 3,481 (19.8) |
| **Age at start of follow-up (years)** |  |  |
| 14-<20 | 5,561 (30.7) | 5,092 (28.9) |
| 20-<22 | 4,188 (23.1) | 3,964 (22.5) |
| 22-<25 | 3,762 (20.7) | 3,648 (20.7) |
| 25-<35 | 3,789 (20.9) | 3,939 (22.4) |
| 35+ | 833 (4.6) | 948 (5.4) |
| **Period of enlistment^a^** |  |  |
| Before World War II | 952 (5.3) | 883 (5.0) |
| During World War II | 5,212 (28.7) | 5,007 (28.5) |
| After World War II | 7,814 (43.1) | 7,689 (43.7) |
| Post-National Service | 4,155 (22.9) | 4,012 (22.8) |
| **Rank at enlistment** |  |  |
| Private | 17,955 (99.3) | 17,428 (99.4) |
| Other | 119 (0.7) | 112 (0.6) |
| Missing | 59 | 51 |
| **Total duration of service (years)** |  |  |
| <2 | 436 (2.4) | 2,785 (15.8) |
| 2<3 | 2,825 (15.6) | 3,407 (19.4) |
| 3<5 | 2,808 (15.5) | 2,722 (15.5) |
| 5<10 | 6,972 (38.6) | 5,559 (31.6) |
| 10+ | 5,028 (27.8) | 3,115 (17.7) |
| Missing | 64 | 3 |
| **Previous duration of service before test/start of follow-up (years)** |  |  |
| <1 | 2,972 (16.4) | 2,927 (16.6) |
| 1<2 | 5,154 (28.4) | 5,040 (28.7) |
| 2<5 | 6,631 (36.6) | 6,299 (35.8) |
| 5<10 | 2,336 (12.9) | 2,282 (13.0) |
| 10+ | 1,040 (5.7) | 1,043 (5.9) |
| **Decade of first test at Porton Down** |  |  |
| 1940s | 6,401 (35.3) |  |
| 1950s | 6,083 (33.6) |  |
| 1960s | 2,251 (12.4) |  |
| 1970s | 1,955 (10.8) |  |
| 1980s | 1,443 (8.0) |  |
| **Vital status** |  |  |
| Deceased | 10,935 (60.3) | 10,658 (60.6) |
| Alive | 5,266 (29.0) | 5,298 (30.1) |
| Lost to follow-up^b^ | 1,932 (10.7) | 1,635 (9.3) |
| **Any neoplasm^c^** |  |  |
| Yes | 5,396 (31.9) | 5,475 (33.1) |
| No | 11,531 (68.1) | 11,058 (66.9) |

**a:** World War II dates taken as 1 September 1939 to 30 April 1945; national service dates taken as 1 May 1945 to 31 December 1960
**b:** last known date alive in the UK e.g., discharge from the services, emigration, or date last traced **c:** Of 16,927 Porton Down veterans and 16,533 non-Porton Down veterans alive at the start of follow-up for cancer (1^st^ January 1971)

**Table S2.** Characteristics of the study sample for cancer analysis comprising 16,927 Porton Down veterans and 16,533 non-Porton Down veterans

|  | **Porton Down veterans** | **Non-Porton Down veterans** |
| --- | --- | --- |
|  | n (%) | n (%) |
| **Service at enlistment** |  |  |
| Army | 10,272 (60.7) | 9,950 (60.2) |
| Airforce | 3,917 (23.1) | 3,903 (23.6) |
| Navy | 2,738 (16.2) | 2,680 (16.2) |
| **Decade of birth** |  |  |
| 1920< | 2,918 (17.2) | 3,075 (18.6) |
| 1920-29 | 3,175 (18.8) | 2,977 (18.0) |
| 1930-39 | 5,943 (35.1) | 5,863 (35.5) |
| >1940 | 4,891 (28.9) | 4,618 (27.9) |
| **Place of birth** |  |  |
| England | 13,367 (79.0) | 12,971 (78.5) |
| Scotland | 1,613 (9.5) | 1,695 (10.3) |
| Wales | 840 (5.0) | 859 (5.2) |
| Northern Ireland | 256 (1.5) | 224 (1.4) |
| Other | 851 (5.0) | 784 (4.7) |
| **Age at enlistment (years)** |  |  |
| <16 | 1,212 (7.2) | 1,200 (7.3) |
| 16-17 | 4,841 (28.6) | 4,213 (25.5) |
| 18-19 | 6,153 (36.4) | 5,942 (35.9) |
| 20-21 | 2,095 (12.4) | 2,233 (13.5) |
| >21 | 2,626 (15.5) | 2,945 (17.8) |
| **Age at start of follow-up (years)** |  |  |
| 14-<20 | 5,358 (31.7) | 4,941 (29.9) |
| 20-<22 | 4,031 (23.8) | 3,816 (23.1) |
| 22-<25 | 3,573 (21.1) | 3,505 (21.2) |
| 25-<35 | 3,360 (19.8) | 3,541 (21.4) |
| 35+ | 605 (3.6) | 730 (4.4) |
| **Period of enlistment** |  |  |
| Before World War II | 736 (4.3) | 703 (4.3) |
| During World War II | 4,412 (26.1) | 4,313 (26.1) |
| After World War II | 7,634 (45.1) | 7,515 (45.5) |
| Post-National Service | 4,145 (24.5) | 4,002 (24.2) |
| **Rank at enlistment** |  |  |
| Private | 16,764 (99.4) | 16,376 (99.3) |
| Other | 109 (0.6) | 109 (0.7) |
| Missing | 54 | 48 |
| **Previous duration of service before test (years)** |  |  |
| <1 | 2,782 (16.4) | 2,744 (16.6) |
| 1<2 | 4,808 (28.4) | 4,753 (28.7) |
| 2<5 | 6,155 (36.4) | 5,882 (35.6) |
| 5<10 | 2,255 (13.3) | 2,201 (13.3) |
| 10+ | 927 (5.5) | 953 (5.8) |
| **Total duration of service (years)** |  |  |
| <2 | 376 (2.2) | 2,638 (16.0) |
| 2<3 | 2,690 (15.9) | 3,281 (19.8) |
| 3<5 | 2,500 (14.8) | 2,456 (14.9) |
| 5<10 | 6,469 (38.4) | 5,161 (31.2) |
| 10+ | 4,833 (28.7) | 2,995 (18.1) |
| Missing | 59 | 2 |
| **Any neoplasm** |  |  |
| No | 11,531 (68.1) | 11,058 (66.9) |
| Yes | 5,396 (31.9) | 5,475 (33.1) |
| **Total number of neoplasms** |  |  |
| 0 | 11,531 (68.1) | 11,058 (66.9) |
| 1 | 4,341 (25.6) | 4,437 (26.8) |
| 2+ | 1,055 (6.2) | 1,038 (6.3) |
| **Vital status** |  |  |
| Deceased | 10,086 (59.6) | 9,897 (59.9) |
| Alive | 5,266 (31.1) | 5,296 (32.0) |
| Lost to follow-up^a^ | 1,575 (9.3) | 1,340 (8.1) |

**a:** last known date alive in the UK e.g. discharge from the services, emigration, or date last traced

**Table S3.** Hazard ratios for the association between attendance at Porton Down and all-cause mortality by period of Porton Down attendance

|  | **Observed deaths (n)** | | **HR (95% CI)** | |
| --- | --- | --- | --- | --- |
| **Period of attendance^a^** | **Porton Down veterans** | **Non-Porton Down veterans** | **Adjusted for age and calendar period** | **Fully adjusted^b^** |
| All | 10,935 | 10,658 | 1.06 (1.03, 1.09) | 1.06 (1.03, 1.09) |
| 1941-44 | 4,580 | 4,358 | 1.05 (1.01, 1.10) | 1.05 (1.01, 1.10) |
| 1945-49 | 1,041 | 1,085 | 1.02 (0.93, 1.11) | 1.01 (0.93, 1.11) |
| 1950-54 | 2,474 | 2,456 | 1.04 (0.99, 1.11) | 1.04 (0.99, 1.10) |
| 1955-59 | 1,387 | 1,329 | 1.09 (1.01, 1.17) | 1.08 (1.00, 1.16) |
| 1960-64 | 625 | 556 | 1.34 (1.19, 1.50) | 1.34 (1.19, 1.50) |
| 1965-69 | 318 | 322 | 1.04 (0.89, 1.22) | 1.05 (0.90, 1.23) |
| 1970-74 | 299 | 315 | 0.94 (0.80, 1.10) | 0.93 (0.79, 1.09) |
| 1975-79 | 96 | 125 | 0.80 (0.61, 1.05) | 0.82 (0.63, 1.08) |
| 1980+ | 115 | 112 | 1.04 (0.77, 1.40) | 1.05 (0.77, 1.41) |

Reference category: ‘Non-Porton Down veterans’
**a:** Year of start of follow-up for non-Porton Down veterans
**b:** Adjusted for branch of service, previous duration of service, and place of birth

**Table S4.** E-value calculations for associations between Porton Down attendance and selected outcomes presented in tables 2, 3 and 4

|  | **Fully adjusted  (HR 95% CI)^a^** | **E-value^b^** | **E-value (CI)^c^** |
| --- | --- | --- | --- |
| **All-cause mortality by period of attendance (from table 2)** |  |  |  |
| All | 1.06 (1.03-1.09) | 1.25 | 1.17 |
| 1941-44 | 1.05 (1.01-1.10) | 1.22 | 1.09 |
| 1955-59 | 1.08 (1.00-1.16) | 1.3 | 1.00 |
| 1960-64 | 1.34 (1.19-1.50) | 1.75 | 1.51 |
| **Cause-specific mortality (from table 3)** |  |  |  |
| Infectious and parasitic (A00-B99) | 1.32 (0.99, 1.78) | 1.97 | 1.00 |
| Malignant neoplasms (All) (C00-97) | 1.05 (1.00-1.10) | 1.22 | 1.00 |
| Lung (C34) | 1.10 (1.01, 1.20) | 1.43 | 1.11 |
| In situ, benign, and unspecified neoplasms (D10-48) | 0.70 (0.49, 1.00) | 2.21 | 1.00 |
| Circulatory system All (I00-99) | 1.06 (1.01, 1.10) | 1.25 | 1.09 |
| Ischaemic heart diseases (I20-25) | 1.08 (1.02, 1.14) | 1.37 | 1.16 |
| Genitourinary system (N00-99) | 1.34 (1.05, 1.70) | 2.02 | 1.28 |
| All external causes (S00-T98, V01-Y98) | 1.15 (1.00, 1.32) | 1.57 | 1.00 |
| Alcohol attributable (F102, K701, K703, K704, K709) | 1.44 (1.07, 1.94) | 2.24 | 1.34 |
| Smoking-related (highly causal only; C33, C34, J40-J44) | 1.07 (1.01, 1.15) | 1.34 | 1.11 |
| **Cause-specific mortality (1960-1964 only) (from table 3)** |  |  |  |
| All-cause | 1.34 (1.19, 1.50) | 1.75 | 1.51 |
| Malignant neoplasms (All) (C00-97) | 1.47 (1.21, 1.78) | 1.94 | 1.54 |
| Oesophageal (C15) | 2.44 (1.04, 5.74) | 4.31 | 1.24 |
| Lung (C34) | 1.68 (1.15, 2.46) | 2.75 | 1.57 |
| Circulatory system All (I00-99) | 1.38 (1.12, 1.69) | 2.1 | 1.49 |
| Ischaemic heart diseases (I20-25) | 1.60 (1.24, 2.06) | 2.58 | 1.79 |
| Smoking-related (highly causal only; C33, C34, J40-J44) | 1.48 (1.09, 2.00) | 2.32 | 1.40 |
| **Cancer incidence (from table 4)** |  |  |  |
| Trachea, bronchus, and lung (C33, C34) | 1.09 (1.00, 1.18) | 1.4 | 1.00 |
| Other skin (C44) | 0.90 (0.82, 0.98) | 1.46 | 1.17 |
| Other urinary tract (C64-C66, C68) | 0.79 (0.62-1.00) | 1.85 | 1.00 |
| All other primary malignant neoplasms | 1.19 (1.00, 1.42) | 1.67 | 1.00 |
| Any neoplasm of uncertain or unknown behaviour (D37-D48) | 1.27 (1.06, 1.54) | 1.86 | 1.31 |

Reference category: ‘Non-Porton Down veterans’
**a:** Adjusted for branch of service, previous duration of service, period of joining, and place of birth
b: E-values represent the minimum strength of association on the risk-ratio scale that an unmeasured confounder would need to have with both attendance at Porton Down and the outcome conditional on the measured covariates, to fully attenuate the association
c: E-values for the limit of the confidence interval closest to the null.

a: During phase two of the study, NHS-CR mortality data was available from 1992 (England and Wales) and 1974 (Scotland) only; for untraced participants, data from the first phase of the study was used if available.

**Supplementary results 1.** Competing risk analysis for selected associations

Competing risk analysis which provided similar but slightly attenuated estimates for key associations. Age and calendar period adjusted results for deaths from circulatory system diseases (sub-distribution hazard ratio (SHR)=1.03, 0.98-1.08), respiratory diseases (SHR=0.97, 0.90-1.05), genitourinary diseases (SHR=1.30, 1.01-1.63) and alcohol attributable deaths (SHR=1.38, 1.03-1.85) were comparable.

**Supplementary results 2.** Associations between type of chemical exposure and all-cause mortality in veterans who attended Porton Down between 1960-1964

Exploratory analyses demonstrated that age-adjusted hazards ratios for all-cause mortality were raised for multiple types of chemical exposures during 1960-1964, i.e. sulfur mustard (n=534; HR=1.37, 1.18-1.59), CS gas (2-chlorobenzalmalononitrile) (n=381; HR=1.36, 1.15-1.63), pralidoxime (n=199; HR=1.21, 0.94-1.54), sarin (n=163; HR=1.36, 1.07-1.73) and CR gas (dibenzoxazepine) (n=162; HR=1.50, 1.17-1.93), compared to non-Porton Down veterans.
